# Hard for humans, hard for machines: predicting readmission after psychiatric hospitalization using narrative notes

**DOI:** 10.1038/s41398-020-01104-w

**Published:** 2021-01-11

**Authors:** William Boag, Olga Kovaleva, Thomas H. McCoy, Anna Rumshisky, Peter Szolovits, Roy H. Perlis

**Affiliations:** 1 Computer Science and Artificial Intelligence Laboratory, Massachusetts Institute of Technology, Cambridge, MA 02139 USA; 2 Department of Computer Science, University of Massachusetts Lowell, Lowell, MA USA; 3 Center for Quantitative Health, Division of Clinical Research, Massachusetts General Hospital, 185 Cambridge Street, Boston, MA 02114 USA

**Keywords:** Psychiatric disorders, Human behaviour

## Abstract

Machine learning has been suggested as a means of identifying individuals at greatest risk for hospital readmission, including psychiatric readmission. We sought to compare the performance of predictive models that use interpretable representations derived via topic modeling to the performance of human experts and nonexperts. We examined all 5076 admissions to a general psychiatry inpatient unit between 2009 and 2016 using electronic health records. We developed multiple models to predict 180-day readmission for these admissions based on features derived from narrative discharge summaries, augmented by baseline sociodemographic and clinical features. We developed models using a training set comprising 70% of the cohort and evaluated on the remaining 30%. Baseline models using demographic features for prediction achieved an area under the curve (AUC) of 0.675 [95% CI 0.674–0.676] on an independent testing set, while language-based models also incorporating bag-of-words features, discharge summaries topics identified by Latent Dirichlet allocation (LDA), and prior psychiatric admissions achieved AUC of 0.726 [95% CI 0.725–0.727]. To characterize the difficulty of the task, we also compared the performance of these classifiers to both expert and nonexpert human raters, with and without feedback, on a subset of 75 test cases. These models outperformed humans on average, including predictions by experienced psychiatrists. Typical note tokens or topics associated with readmission risk were related to pregnancy/postpartum state, family relationships, and psychosis.

## Introduction

The ability of prediction tools based on machine learning (ML) to identify high-risk individuals among clinical populations has been embraced across health care^1^. In psychiatry, a number of reports suggest the ability of these approaches to improve on chance in predicting hospital readmission, suicide attempts, or mortality, for example^2,3^. In previous work, we demonstrated that incorporation of features based on topic modeling of narrative notes improved prediction among individuals with major depressive disorder. This approach has the important feature of yielding topics that may be more interpretable than individual words or codes^4,5^.

However, several important questions remain unanswered by prior work. First, most prior investigations focused on a single, homogeneous patient group. In real-world implementation, clinical populations are rarely so selected, which may pose challenges for natural language processing methods. In particular, how well could such models account for populations that are both medically and psychiatrically complex? Second, while prior work indicates improvement over chance, a more relevant question is performance compared to human experts: how well do clinicians perform on a given task, and can ML outperform human learning?

To address these concerns, we drew on 8 years of electronic health records from a large inpatient psychiatric unit. We applied standard ML approaches and then compared these approaches to expert and nonexpert clinical annotation, as a means of understanding whether ML models could equal or surpass human prediction.

## Materials and methods

### Study design and cohort generation

We utilized a retrospective cohort design, drawing from the electronic health records of a Boston academic medical center’s inpatient psychiatric unit. All consecutive admissions between January 1, 2009 and December 31, 2016 were included. Data were extracted and stored using i2b2 server software^6^; coded features extracted included age, sex, race/ethnicity, insurance type, as well as admission diagnoses, with ICD9 codes mapped to the HCUP/CCS Level 2 ontology^7^. The data mart also included all narrative discharge summaries. In total, we analyzed 5076 health records, 75 of which were randomly sampled from the test split for our human prediction study. The primary outcome of interest was all-cause hospital readmission within 180 days, recognizing that psychiatric illness contributes to adverse outcomes that may not be fully captured by psychiatric hospitalization.

The Partners HealthCare Human Research Committee approved the study protocol. As no participant contact was required in this study based on secondary use of data arising from routine clinical care, the committee waived the requirement for informed consent as detailed by 45 CFR 46.116.

### Natural language processing of discharge summaries

**Fig. 1:**
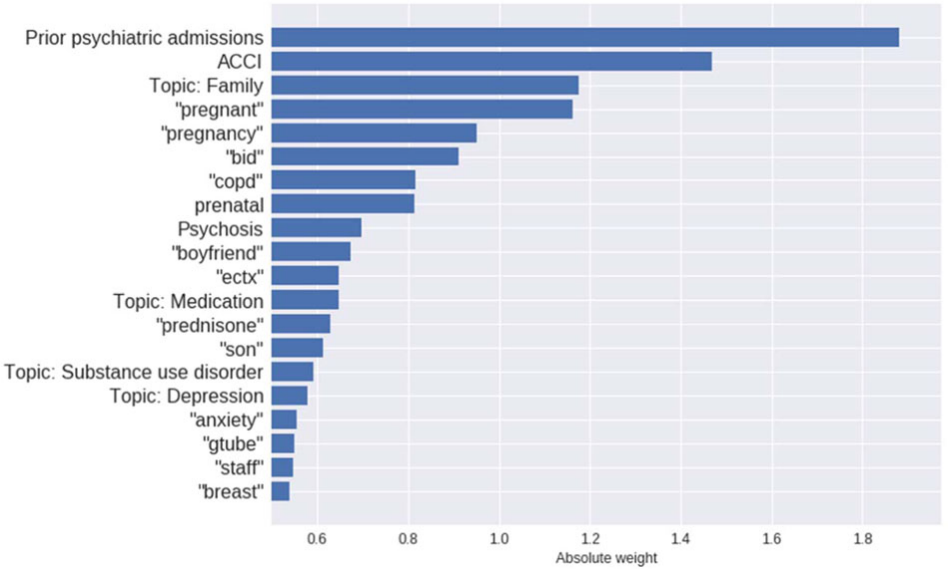
Weights for most important features in logistic regression model.

We preprocessed the raw text of the records by lowercasing the words, removing the punctuation, and replacing all numbers with a placeholder “NUM” token. The text was split into tokens with the regex-based tokenizer of the nltk library v3.2.5^8^, with whitespace characters serving as token delimiters. The resulting vocabulary consisted of 20878 tokens. Length distribution of the discharge summaries is summarized in [Supplement A Fig. 1]. After text preprocessing, we represented the input discharge narratives using several sets of features which are described below. No attempt was made to distinguish discharge summary sections, as note format varied substantially in structure.

#### TF-IDF features

Term frequency-inverse document frequency scoring^9^ allows for weighting every word of the given document by its importance relative to the entire corpus. We used the scikit-learn library v0.20.2^10^ to convert every document to a distribution of scores over a pruned vocabulary. The pruning procedure included filtering out common English stopwords, setting the maximum document frequency to 90% of all documents and the minimum document frequency to 10, based on prior work with psychiatric discharge summaries and visual inspection of distributions. This filtering procedure aims to remove noise such as typographic errors in the narrative notes. As a result, the size of the initial vocabulary was reduced to 11062 unique tokens.

#### LDA features

Latent Dirichlet Allocation^11^ is an unsupervised generative statistical model that uses word co-occurrence to derive a representation for each input document. The derived representation takes the form of a distribution over a predefined number of topics, with each topic corresponding to a distribution over a set of words. In our experiments, we used the gensim library v3.7.0^12^ that uses raw word counts as input to compute the topic distributions. We varied the number of topics from 25 to 100, selected a priori based on prior work with narrative clinical notes to balance overfitting risk without yielding overly sparse topics, and, based on the held-out validation dataset, found the optimal number to be 75 topics (see [Supplement B Tables 1 and 2]). Stability of Supplement B results was confirmed via multiple runs. The resulting 75-dimensional vectors (henceforth, LDA-75) were used as input to the classifier models. Sample derived topics are shown in Supplement C Table 3.

### Prediction models

All models incorporated baseline sociodemographic and clinical features, defined as age at admission, gender, self-reported race (coded as White, African-American, Asian, unknown, and other), age-adjusted Charlson comorbidity index (ACCI)^13,14^, and insurance type (coded as public payer, public Medicaid, public Medicare, and private payer).

Out of the entire cohort, four patients had missing values for age and ACCI index. These missing values were imputed with the corresponding averages over the dataset and the resulting numeric features were further normalized to fall in the range between zero and one. Categorical features were encoded using the one-hot encoding scheme (i.e., presence/absence of a feature). This procedure resulted in a 13-dimensional demographic baseline feature vector per admission. Additionally, the number of prior psychiatric admissions (PrPA) within the past 12 months was used as a predictive feature.

Using the representation described above, we trained support vector machines with linear kernel (SVM) for different feature combinations, including demographics, TF-IDF, LDA-75, demographics+TF-IDF, demographics+LDA, demographics+TF-IDF + LDA, and demographics+TF-IDF + LDA + PrPA. We experimented with other simple ML models (including logistic regression (LR), gradient boosting (XGB), multilayer perceptrons (MLP)), but all models had very similar levels of performance.

In addition to these standard ML algorithms, in preliminary experiments, we also examined two recurrent neural networks: a standard long short-term memory (LSTM) recurrent neural network^15^ and a hierarchical LSTM with attention to model a sequence of sentences where each sentence is a sequence of words^16^. However, due to notably large input size of the tokenized documents, the selected LSTM-based architectures struggled to encode all the relevant information in single fixed-length vectors, leading to consistently poor performance even with optimization of hyperparameters.

### Comparison with human prediction

We sought to understand the difficulty of the prediction task for human raters, including both clinical experts and individuals without domain-specific knowledge. To achieve this objective, we used two sets of human raters (*n* = 7 total). The first consisted of three psychiatrists with at least 5 years of clinical experience, including work on inpatient units and consult-liaison psychiatry. The second consisted of four graduate students without formal postgraduate training in psychiatry or psychology. Human raters were given access to the full discharge summary, as well as the structured demographics information, but were blinded to model features including prior psychiatric admissions (PrPA). Each rater began by scoring the same 25 records, randomly selected to include 9 readmissions and 16 non-readmissions within 180 days. Each record was scored in terms of likely readmission^17^ and the associated judgment confidence. In order to establish a baseline for prediction accuracy, no feedback was provided for these predictions. In the second phase (henceforth, learning phrase), participants scored a set of additional 50 randomly sampled records (with the same ratio of readmission to non-readmission), receiving feedback about the correct answer after each prediction. These additional 50 records were the same for all human raters, and were shown in the same order.

## Results

**Fig. 2:**
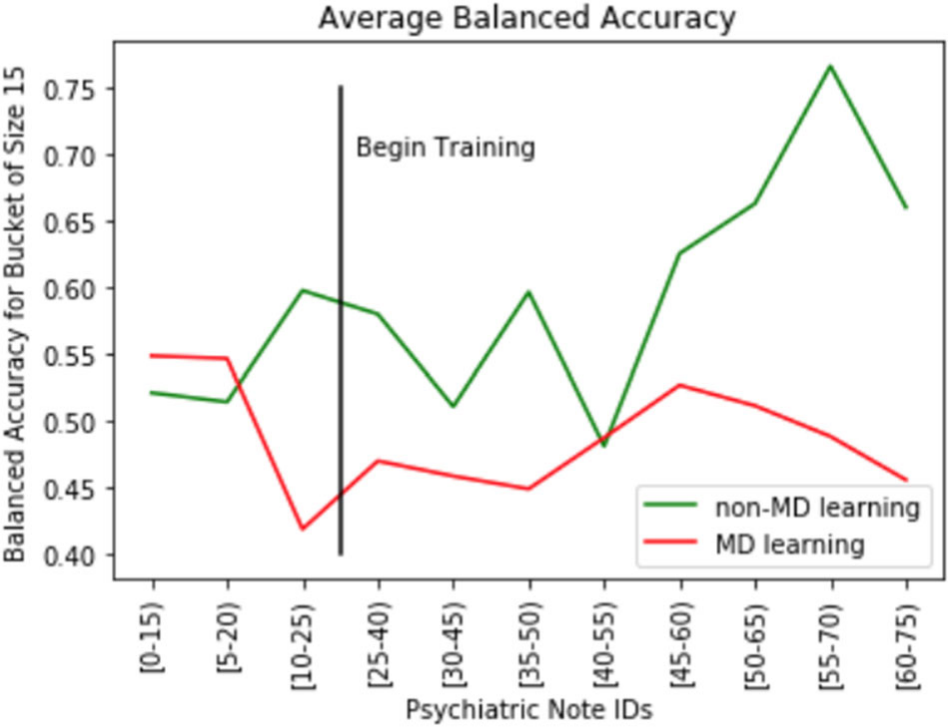
Change in human performance over time. As all raters were shown the same notes in the same order, the figure also enables a comparison of how well each group was able to improve their performance over time. In particular, the nonclinicians were able to improve their performance substantially upon receiving feedback, with a ~10% increase in absolute accuracy between their initial baseline performance and their final set of predictions after training. Performance of expert annotators (i.e., psychiatrists), on the other hand, did not improve notably over time.

Characteristics of the full cohort (*n* = 5076 psychiatric admissions), as well as the training (70%) and testing (30%) cohorts stratified by sex, age, ACCI score, and number of prior admissions, are presented in Table 1. The cohort included a broad range of admission diagnoses, with 44 unique CCS categories in total; the most common diagnosis was mood disorder (5.8.657). The full distribution of the top 10 most frequent admission CCS codes can be found in [Supplement C Fig. 2]. Out of the 5076 patients, 1168 were readmitted within 180 days (23%).

**Table 1:** Population characteristics.

Variable	Value	Train	Test
Count	3553	1523	
Gender	F	1781 (50.1%)	771 (50.6%)
	M	1772 (49.9%)	752 (49.4%)
Prior admissions		2.3 [4.5]	2.2 [4.0]
ACCI		3.7 [4.5]	3.8 [4.6]
Age		45.0 [16.7]	45.2 [16.6]
Median income of ZIP code		65557 [23427]	65679 [23869]
Insurance	Private	2268 (63.9%)	970 (63.7%)
	Public	1285 (36.1%)	553 (36.3%)
180-day readmission	Y	819 (23.1%)	349 (22.9%)
	N	2734 (76.9%)	1174 (77.1%)

ACCI is Age-adjusted Charlson Comorbidity Index. Parenthetical numbers for categorical variables denote % membership. Bracketed numbers for continuous variables denote the standard deviation. Note that prior admissions statistics refer to prior general (rather than psychiatric) admissions.

The bold font corresponds to the best performing model for a given set of features (in the leftmost column).

## Machine learning

The training set was randomly split into 10 folds to perform cross-validation, which was carried out separately with each of the models (SVM, LR, XGB, and MLP) for the feature representations listed in Methods section above. Using random search over hyperparameter space, we picked the best model hyperparameters based on the average score over the folds. To ensure the reproducibility of the results, we repeated the cross-validation procedure 100 times resulting in 100 best hyperparameter configurations. We tested the resulting 100 fine-tuned models, and we report the average test AUC scores with the corresponding confidence intervals in Table 2.

**Table 2:** Overall performance of the tested models.

	LR	SVM	XGB	MLP
Demographics	0.668 [0.665–0.671]	0.659 [0.658–0.660]	**0.675 [0.674–0.676]**	0.655 [0.652–0.658]
TF-IDF	0.682 [0.678–0.686]	**0.692 [0.691–0.693]**	0.673 [0.670–0.676]	0.630 [0.620–0.640]
LDA-75	0.661 [0.660–0.662]	**0.663 [0.662–0.664]**	0.634 [0.633–0.635]	0.649 [0.646–0.652]
Demographics + TF-IDF	0.687 [0.679–0.695]	**0.698 [0.697–0.699]**	0.688 [0.687–0.689]	0.631 [0.621–0.641]
Demographics + LDA	**0.685 [0.684–0.686]**	**0.685 [0.684–0.686]**	0.679 [0.677–0.681]	0.643 [0.628–0.658]
Demographics + TF-IDF + LDA-75	0.696 [0.695–0.697]	**0.701 [0.699–0.703]**	0.688 [0.687–0/689]	0.626 [0.607–0.645]
Demographics + TF-IDF + LDA-75 + PrPA	**0.726 [0.725–0.727]**	0.721 [0.720–0.722]	0.706 [0.707–0.705]	0.628 [0.612–0.644]

The bold font corresponds to the best performing model for a given set of features (in the leftmost column).

To identify the most relevant features in ML models and facilitate interpretation, we extracted the top 20 weights (sorted by their magnitude) assigned by the best logistic regression model (with the AUC of .726). The histogram suggests that prior psychiatric admissions, single tokens (pregnancy-related terms; family relationships) as well as age-adjusted Charlson comorbidity index represent major predictors of readmission (Fig. 1). Topics provide additional predictive support, most notably capturing family relationships (Topic 13) and psychosis and antipsychotics (Topic 44).

## Human learning

Since human raters did not have access to the information about prior psychiatric admissions, we focused on the SVM results, which yielded the best performance for the demographics+TF-IDF + LDA-75 feature set. Table 3 compares prediction results for 180-day readmission for the SVM models and human raters. Since AUC requires ranked scores (and therefore cannot be reported for human judgments), we report F1, accuracy, and balanced accuracy. Also, for the purposes of comparison, the figures reported for ML models come from a single run, rather than averaged over multiple runs. We also report the baseline performance for two arbitrarily-selected predictors to capture the recurrent nature of psychiatric illness: (a) always predicting that the patient will be readmitted, and (b) always predicting that the patient will be readmitted if there are 5 or more prior psychiatric admissions in the past 12 months.

**Table 3:** Performance comparison between humans and predictive models on the full test set (*n* = 1523) and the human-annotated learning-phase subset (*n* = 50).

Paradigm	Method	Accuracy (*n* = 50)	Balanced Accuracy (*n* = 50)	F1 (*n* = 50)	F1 (*n* = 1523)	AUC (*n* = 1523)
Human	Average non-MD	0.655	0.635	0.527	—	—
Human	Average MD	0.527	0.484	0.306	—	—
Baseline	Always readmit	0.280	0.500	0.437	0.372	—
Baseline	5 or more admissions in 12 months	0.640	0.706	0.571	0.432	—
ML	SVM (Demographics)	0.680	**0.770**	**0.634**	0.414	0.658
ML	SVM (Demographics + TF-IDF)	0.600	0.665	0.533	0.430	0.698
ML	SVM (Demographics + LDA)	**0.700**	0.704	0.571	0.436	0.685
ML	SVM (Demographics + TF-IDF + LDA)	0.620	0.651	0.522	**0.449**	**0.703**

Bold entries represent the best performing model (or human category) for each of the target metrics given in the column headers.

Overall, all of the ML models, including very basic ones, outperformed the average human performance (balanced accuracy of 0.484 for expert and 0.635 for nonexpert human judges, respectively). Note that the performance of the ML models on the human-annotated learning-phase data (*n* = 50) is higher than on the full test set (*n* = 1523), with F1 scores of 0.52–0.63 and 0.41–0.45, respectively. Also, for this smaller subset, models based on the minimal baseline demographic features outperformed the models using NLP-derived features. This suggests that the human-annotated subset was not entirely representative of the full test set, which is in part due to the small sample size.

We also looked at human performance over time, both with and without feedback on whether the generated predictions were correct. Figure 2 illustrates the average performance for expert (MD) and nonexpert (non-MD) annotators; each timestep of the *x*-axis indicates a set of 15 predictions (i.e., 15 hospital discharges) grouped together using a sliding window with a 5-note stride. The first three timesteps (covering notes 0–25) indicate human performance before receiving any feedback. Starting from the 25–40 window, we can see performance over time.

## Discussion

In this investigation of more than 5000 admissions to a psychiatric inpatient unit, we found that prediction models incorporating topics derived from Latent Dirichlet Allocation in addition to simpler bag-of-words features performed comparably to, or better than, the models relying solely on bag-of-words or on coded data. These findings are consistent with our own and other prior work^5^; the explanatory power of a single engineered feature, namely prior admissions, is also unsurprising as a marker of illness chronicity and severity. The topics themselves were notable for coherence in capturing predominantly comorbidities (e.g., orthopedic injury), psychosocial features (e.g., family relationships, homelessness), or symptoms (e.g., psychosis, substance abuse).

Conversely, the performance of the models we present is markedly poorer than that of many models predicting medical readmission^1^. We would underscore that this likely reflects the challenging nature of the task; indeed, clinical features strongly predictive of readmission remain unclear, and there are no validated biomarkers or markers of disease progression.

Although the models we developed improve substantially upon chance, we also sought to understand their ability to improve upon prediction by clinicians and nonclinicians. This task allows us to ground these models using a more understandable frame of reference. We observed that ML models outperformed our human raters, especially domain experts, suggesting both the difficulty of the task and that the models are not overlooking obvious features that would improve prediction. The success of the models suggests that there is some additional signal in the notes not captured fully by human raters, potentially identified by aggregating many small indications. Still, the modest performance overall suggests that the upper bound of performance is probably far below “perfect,” reflecting an element of stochasticity of readmission. Of course, neither human nor machine models have access to information not contained in notes that could be highly relevant to readmission risk, such as undocumented aspects of psychosocial circumstances or comorbidities not addressed during an acute hospitalization.

Perhaps more surprisingly, nonclinician raters outperformed clinicians, which may reflect that this task is distinct from standard clinical practice. Further, as the nonclinician raters were better able to improve their performance with feedback, it may be the case that nonexperts are more easily able to conform to new tasks because they have fewer incorrect priors, whereas experts are harder to shift from their existing frameworks.

This latter set of observations is consistent with decades of prior evidence that clinician predictions, when compared to real-world outcomes, often do not substantially exceed chance. In psychiatry, a seminal paper by Pokorny found that even incorporating a range of rating scales did not yield meaningful prediction^18^.

Our results also hint at an opportunity to improve prediction in clinical settings by presenting observed outcomes to clinicians in an iterative process. An important question to consider will be whether ML models can be applied to accelerate or enhance this learning process, by highlighting key topics or terms to consider, for example.

We note several important limitations in considering our results. First, they reflect a single inpatient unit, albeit a large and heterogeneous one. The extent to which results generalize to other health systems remains to be established; it is possible that institution-specific models will be required given the heterogeneity of documentation across institutions. Institutional restrictions on accessing large clinical corpora continue to inhibit work in this area and underscore the need for more cross-institution collaboration.

Second, our prediction task likely underestimates the skill of clinicians in practice, who are able to draw on clinical impressions of patients that may not be fully captured in narrative notes. This distinction likely explains the inability to distinguish trained clinicians from graduate students. Moreover, as we use notes generated at time of hospital discharge, we expect that clinicians do not fully document the extent of concern for readmission, insofar as individuals thought to be at high risk would not be candidates for discharge in the first place.

Still, taken together, our results suggest that ML approaches incorporating topic models consistently exceed clinicians’ ability to predict hospital readmission, while retaining inspectability. While such models are far less performant than some recent efforts in more homogenous datasets, this likely reflects the challenging nature of the task itself. As better approaches to staging or characterizing progression emerge, including biomarkers or clinical metrics, we anticipate that the models we describe will provide a valuable baseline to be improved upon in subsequent work.

## Supplementary information

Supplemental Material

Supplemental Material D, Table 4
