# Supplementary material for: Hard for humans, hard for machines: predicting readmission after psychiatric hospitalization using narrative notes
Source: Transl Psychiatry. 2021 Jan 11;11:32. doi: 10.1038/s41398-020-01104-w (PMC7801508; doi:10.1038/s41398-020-01104-w)
Supplement: Supplementary file 1 — Supplemental Material [file 41398_2020_1104_MOESM1_ESM.docx]

**Supplement A**

The resulting training and testing splits of the discharge summaries have similar distributions of the word and the sentence counts. A median-length record is a fairly long, consisting of 3300 words and 130 sentences.

**Figure 1.** Distribution of word (top row) and sentence (bottom row) counts over the train and test dataset splits.


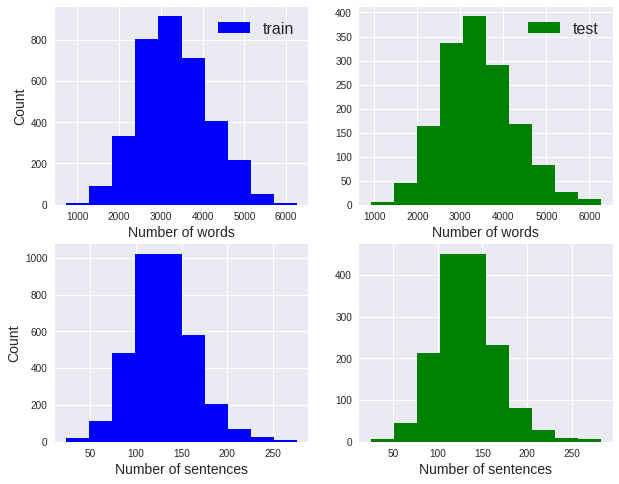


**Supplement B**

Tables 1 and 2 show the results of experiments conducted to optimize the number of topics on a held-out set of the data. 75-topic configuration showed minor performance gain across the board and was selected as the default LDA feature extraction method.

**Table 1.** LDA number of topics experiment (LDA features only). Numbers represent the AUC score achieved on the held-out data. The tested models are logistic regression (LR), support vector machine classifier (SVM), gradient boosting (XGB), multilayer perceptron (MLP).

|  | LR | SVM | XGB | MLP |
| --- | --- | --- | --- | --- |
| 25 topics | 0.65 | 0.65 | 0.65 | 0.65 |
| 50 topics | 0.67 | 0.67 | 0.66 | **0.67** |
| 75 topics | **0.69** | **0.68** | **0.67** | **0.67** |
| 100 topics | 0.65 | 0.66 | 0.64 | 0.65 |

**Table 2.** LDA number of topics experiment (LDA + baseline features). Numbers represent the performance on the held-out data.

|  | LR | SVM | XGB | MLP |
| --- | --- | --- | --- | --- |
| 25 topics | 0.68 | 0.68 | 0.68 | **0.67** |
| 50 topics | 0.69 | 0.69 | **0.69** | **0.67** |
| 75 topics | **0.70** | **0.70** | **0.69** | **0.67** |
| 100 topics | 0.68 | 0.68 | **0.69** | 0.66 |

**Supplement C**

**Table 3.** Examples of more coherent LDA topics

| Topic 8 | abdomen | heart | cardiovascular | motion | ent | musculoskeletal |
| --- | --- | --- | --- | --- | --- | --- |
| Topic 10 | devil | god | decanoate | devils | haldol | cogentin |
| Topic 11 | pregnancy | pregnant | invega | ob | im | prenatal |
| Topic 13 | parents | school | sister | friends | wife | boyfriend |
| Topic 23 | parkinson | carbidopa | levodopa | ect | disease | take |
| Topic 24 | dental | tooth | teeth | caries | political | extraction |
| Topic 36 | fracture | ankle | fractures | orthopedics | parnate | orthopedic |
| Topic 38 | dependence | seizure | overdose | withdrawal | drinking | seizures |
| Topic 44 | risperidone | staff | schizoaffective | schizophrenia | risperdal | clozapine |
| Topic 56 | methadone | wrist | heroin | burn | laceration | dependence |
| Topic 57 | catatonia | ativan | lesions | extremities | stiffness | chills |
| Topic 62 | ms | heroin | police | seroquel | marijuana | cannabis |

The distribution of the admission codes shows the diversity of the investigated cohort.

**Figure 2.** Admission diagnoses codes distribution. The top-10 most frequent codes are shown.


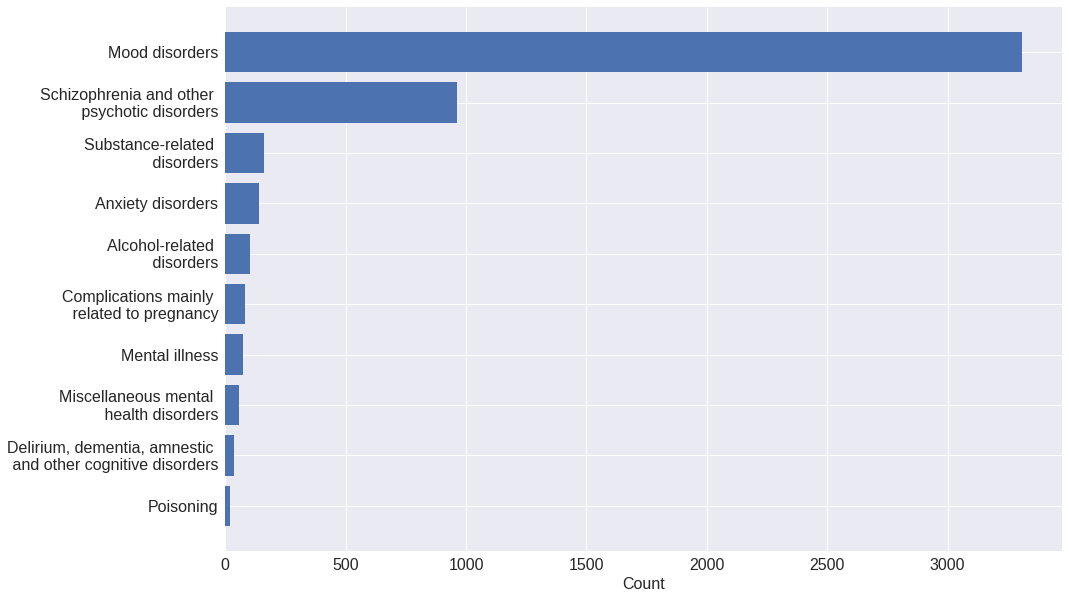


**Supplement D**

**Table 4.** Full list of topics detected by LDA. Each topic consists of a set of words sorted by their scores.

*Please find Supplement D Table 4 in a separate attached Excel file.*
